# Measuring Disability and Its Predicting Factors in a Large Database in Taiwan Using the World Health Organization Disability Assessment Schedule 2.0

**DOI:** 10.3390/ijerph111212148

**Published:** 2014-11-25

**Authors:** Wen-Chou Chi, Kwang-Hwa Chang, Reuben Escorpizo, Chia-Feng Yen, Hua-Fang Liao, Feng-Hang Chang, Hung-Yi Chiou, Sue-Wen Teng, Wen-Ta Chiu, Tsan-Hon Liou

**Affiliations:** 1 Department of Physical Medicine and Rehabilitation, Shuang Ho Hospital, Taipei Medical University, Taipei 235, Taiwan; E-Mail: y6312002@gmail.com; 2 Department of Physical Medicine and Rehabilitation, Wan Fang Hospital, Taipei Medical University, Taipei 116, Taiwan; E-Mail: chang2773@gmail.com; 3 Graduate Institute of Injury Prevention and Control, Taipei Medical University, Taipei 110, Taiwan; E-Mails: fhchang@bu.edu (F.-H.C.); wtchiu.tmu@gmail.com (W.-T.C.); 4 Department of Rehabilitation and Movement Science, College of Nursing and Health Sciences, University of Vermont, Burlington, VT 05401, USA; E-Mail: escorpizo.reuben@gmail.com; 5 Swiss Paraplegic Research, Nottwil 6207, Switzerland; 6 Department of Public Health, Tzu Chi University, Hualien 970, Taiwan; E-Mail: mapleyeng@gmail.com; 7 Chinese Association of Early Intervention Profession for Children with Developmental Delays, Hualien City 970, Taiwan; E-Mail: hfliao@ntu.edu.tw; 8 School and Graduate Institute of Physical Therapy, College of Medicine, National Taiwan University, Taipei 106, Taiwan; 9 School of Public Health, Taipei Medical University, Taipei 116, Taiwan; E-Mail: hychiou@tmu.edu.tw; 10 Ministry of Health and Welfare, Taipei 115, Taiwan; E-Mail: nhswteng@mohw.gov.tw; 11 Department of Physical Medicine and Rehabilitation, College of Medicine, Taipei Medical University, Taipei 116, Taiwan

**Keywords:** disability, ICF, impairment

## Abstract

The definition of disability had been unclear until the International Classification of Functioning, Disability, and Health was promulgated in 2001 by the World Health Organization (WHO). Disability is a critical but relatively neglected public-health concern. We conducted this study to measure disabilities by using the WHO Disability Assessment Schedule 2.0 (WHODAS 2.0) and identify the factors that contribute to disabilities. We obtained and analyzed the data on people who applied to Taiwan’s disability registration system between September 2012 and August 2013. A total of 158,174 cases were selected for this study. Among the people included in this study, 53% were male, and the females were on average 3 years older than the males. More males than females were of a low socioeconomic status, but the rate of employment was higher among the males than among the females. Age, sex, place of residence, and types and severity of impairment were all determined to be factors that independently contributed to disability. This study has demonstrated that disability can be measured and compared using WHODAS 2.0. Increasing the public-health attention devoted to disability and identifying the factors associated with disability can promote independence and social participation in people with disabilities.

## 1. Introduction

Disability has been described as the result of interaction between an individual’s functional impairments, activity limitations, participation restrictions and their environment and personal factors that lead to serious impact on the individual and society [1]. People with disabilities, such as individuals with stroke, hearing impairment, or schizophrenia, often experience challenges in their daily lives, and thus their activities and social participation are limited. Moreover, people with disability account for 15% of the estimated global population of 1 billion [2]. The number of people with disabilities has increased every year in Taiwan from 861,030 (3.8% of the total population) in 2003 to 1,100,436 (4.6% of the total population) in 2011. Disability is one of the most crucial concerns among non-communicable diseases identified by the World Health Organization (WHO) post-2015 agenda [3]. Today, disability is recognized as a critical but neglected public-health concern [4,5,6].

The definition of disability has been debated among experts in the medical and social science fields for decades [1,7]. From the traditional biomedical perspective, disability was considered narrowly to be related to the impairment of body structures or function [8]. However, this perspective ignores the interaction between persons and the environment, which can support or impede a person from being involved in societal roles. In 2001, the World Health Organization (WHO) published the International Classification of Functioning, Disability, and Health (ICF), which promotes a new vision of health and disability and defines disability as a ‘‘difficulty in functioning at the body, person, or societal levels, in one or more life domains, as experienced by an individual with a health condition in interaction with contextual factors’’ Health conditions can vary from neurological disorders, orthopedic injuries, developmental disorders, or psychiatric diseases [9].

Disability is a serious health and societal issue across persons’ lifespan, and its associated healthcare and social welfare expenditures can be enormous to society [10]. Therefore, it is important to identify the attributors of disability and the needs for resource allocation. The Global Burden of Disease report has identified five leading diseases contributing to disability: eye diseases, hearing loss, dementia, musculoskeletal diseases, and heart disease [11]. However, this report has been criticized for failing to use an ICF-based, standardized disability measure that acknowledges contextual influence rather than using diagnoses to determine disability. Recognizing this issue, Sousa *et al.* used a standardized disability assessment tool, the WHO Disability Assessment Schedule 2.0 (WHODAS 2.0), to investigate the contribution of chronic diseases to disability in elderly people in low- and middle-income countries [12]. WHODAS 2.0 is a generic instrument developed in parallel with the ICF to assess levels of functioning [4,13]. This disability instrument treats all disorders at parity in assessing levels of function, and has demonstrated rigorous validity, reliability, and cross-culture applicability in more than 30 languages [14,15,16,17]. Using WHODAS 2.0 in a cross-country survey, Sousa *et al.* found dementia as the most crucial independent contributor to disability [12]. Nevertheless, the study has a limited focus in the older population with lower incomes, so the findings may not be generalized to a broader population.

To identify the attributors of disability across ages and income-levels, a population-based research with a broader sample is needed. The present study used a population-based dataset collected in Taiwan to identify the contribution to disability. The specific study aims included: (1) to examine the associations between disability and impairment types and severity; (2) to identify the factors that contribute to disability.

## 2. Methods

### 2.1. Development of Disability Registration System in Taiwan

Since 1980, the Taiwanese government has enacted legislative procedures to create and revise disability categories. A person who meets the criteria required to be eligible for disability benefits might be granted financial aid and in-kind benefits from the government. However, the criteria used for evaluating disabilities were based mainly on the medical model in which, before 2007, disability was considered to be just either a physical or mental impairment. Thus, physicians identified candidates who were eligible for disability benefits based mainly on the severity of bodily impairment, but did not adequately evaluate the person’s daily activity and social participation or the environmental factors that affected these individuals. In line with the United Nations Convention on the Rights of Persons with Disabilities, Taiwan legislated a constitutional amendment known as the People with Disabilities Rights Protection Act in 2007 [18,19]. The act has mandated that a person’s eligibility for disability benefits should be assessed based on the ICF framework starting from July 2012. This nationwide initiative has been developed to promote social participation of people with disabilities and form links between disability evaluation, needs assessment, and social-welfare services available for people with disabilities.

The preparation for reforming the disability system began in 2007; the activities that were involved in the three main phases developed for the purpose of reaching the specific aims are described in detail elsewhere [18,19]. Under this new system, 236 hospitals throughout Taiwan are authorized to evaluate disability, and in these hospitals, physicians who are specialists in distinct areas perform the medical evaluations. Moreover, between 2010 and 2013, a total of 7125 clinicians including physical therapists, occupational therapists, social workers, psychologists, and senior nurses who have cared for people with disabilities for at least one year from these hospitals were trained to be qualified for the evaluation. Those clinicians must receive a 2-day on-job training for the evaluation. In most situations, one physician gives the ICD-9-CM diagnosis codes and performs the medical assessment, one qualified clinician does the functional assessment. After the two assessments, a medical evaluation report with the information of disability determination, type of impairment and severity of impairment will be finished. The criteria for eligibility were set by our experts’ consensus according to the definition of the ICF qualifier.

### 2.2. Data Source

In July 2012, the Ministry of Health and Welfare in Taiwan established in collaboration with researchers and social welfare groups, a registry system for disability evaluation, functional assessment, and provision of social-welfare services based on the ICF framework [20]. We applied the data to the authority, the Social and Family Affairs Administration, Ministry of Health and Welfare, and obtained anonymized data with permission. The Joint Institutional Review Board at Taipei Medical University has approved this study (Approval No. 201004001 and No. 201205042).

### 2.3. Case Selection

We obtained data on people who applied under the new disability system between September 2012 and August 2013, and we included people who finished the entire evaluation procedure and were eventually provided disability benefits by the government of Taiwan. We excluded people under 18, those who were deemed ineligible for disability evaluation, people with data that were inadequate and were not available, and those with coexisting diseases.

### 2.4. Personal Profiles

The data obtained from the registry were sorted into three sections. The first section recorded sociodemographic information such as age, sex, residence area, education level, ethnicity, household income, work status, and family type. The second section consisted of medical reports, which contained disability-related details such as onset of disease, cause of disability, diagnosis (ICD-9CM), and body functions (b codes) and structural components (s codes) defined by the ICF. The severity of impairment was determined based on the highest qualifier of b or s codes (1 = mild: 5%–24% impairment, 2 = moderate: 25%–49% impairment, 3 = severe: 50%–95% impairment, 4 = complete: 96%–100% impairment); for example, if an individual with schizophrenia received an ICF coding of b110.2, b122.3, and b167.1, the severity of impairment was determined to be 3, the highest qualifier (which indicates a severe case). In the third section, we recorded the functional score of disability evaluation system (FUNDES) that included WHODAS 2.0 (36 items) of traditional Chinese version [18,19,21].

### 2.5. Causes of Disability

Based on physicians’ diagnoses of disabilities within the registry, we identified 10 leading causes of disability that were included under these ICD-9CM groupings: schizophrenia (295), stroke (431–438), spinal cord injury (344.0, 344.1, 767.4, 806, 907.2, and 952), hearing impairment (388–389), dementia (290 and 331), bipolar affective disorder (296, except for 296.2 and 296.3), visual impairment (360–379), mental retardation (317–319), depression (296.2–296.3), and autism (299). These 10 causes of disability were categorized into three types of impairment: sensory impairment, which included visual and hearing diseases; physical impairment, which included stroke and spinal cord injury; and mental impairment, which included schizophrenia, dementia, depression, bipolar affective disorder, mental retardation, and autism. In this study, we use impairment as a negative aspect of body function and structure, where disability serves as an umbrella term for impairments, activity limitations or participation restrictions.

### 2.6. Domain and Summary Scores of WHODAS 2.0

We used the 36-item WHODAS 2.0 questionnaire, which was administered by interviewing the participants or their proxies if the participants could not answer the questions. Participants were asked to consider how much their disabilities interfered with their lives in the last 30 days and then answered on a 5-point response scale from 1 (none) to 5 (extreme/cannot do). The domain scores and summary score (the general-disability latent variable) were calculated using the 36-item version of WHODAS 2.0 if the participants were employed or were students, and the 32-item version (which lacks the four items related to work ability) if individuals were unemployed; the scoring algorithm that we used is available through the WHO. All scores ranged from 0 (best) to 100 (worst), and high scores indicated a high level of disability. The score computation allowed for up to 30% of items to be missing per domain, and substitution of mean (by domain) was used for the imputation of missing data. The WHODAS 2.0 consists of six domains: understanding and communicating, getting around, self-care, getting along with others, life activities, and participation in society. Currently, WHODAS 2.0 is used only for people over 18; another version of WHODAS is underdeveloped for evaluating disabilities in children.

### 2.7. Statistical Analysis

Statistical analyses were performed using SAS, Version 9.2 (SAS Institute Inc., Cary, NC, USA). Chi-square tests were used in the analyses. To evaluate the association between impairment and WHODAS 2.0 scores, we constructed a Poisson regression model to assess the odds ratio between WHODAS 2.0 scores and control for demographic variables (such as sex, age, and educational level). We considered *P* < 0.05 to be statistically significant.

## 3. Results

### 3.1. Sample Size

**Figure 1:**
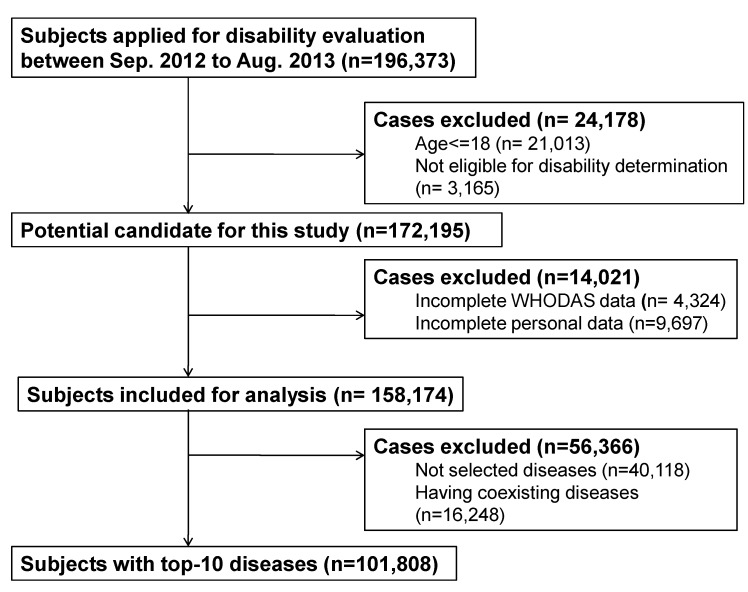
Case selection flowchart.

After excluding the ineligible cases, we selected 158,174 cases for this study (Figure 1). The demographic and basic characteristics of the study participants were listed in Table 1. The female participants were on average 3 years older than the males, and nearly half of the participants lived in urban areas. More males than females belonged to a low socioeconomic status, but the employment rate was higher among males than among females.

**Table 1:** Basic characteristics of study participants.

Variables (n = 158,174)		Female (n = 77,736)	Male (n = 80,438)	*P* Value *
**Age** (year ± SD)		59.35 ± 18.33	56.27 ± 18.09	<0.001
**Residence**				<0.001
Urban		33,185 (46.30%)	35,131 (43.70%)	
Suburban		23,082 (32.20%)	26,825 (33.40%)	
Rural		15,468 (21.60%)	18,478 (23.00%)	
**Education**				<0.001
	No education	8880 (25.36%)	2802 (7.13%)	
	Primary school	9915 (28.31%)	10,745 (27.36%)	
	Above primary school	16,225 (46.33%)	25,730 (65.51%)	
**Socioeconomic status**				<0.001
Normal (≥6122 USD/year)		18,545 (91.30%)	21,224 (89.80%)	
Middle-low (4082~6122 USD/year)		804 (4.00%)	910 (3.90%)	
Low (<4082 USD/year)		973 (4.80%)	1497 (6.30%)	
**Work status**				<0.001
Employed		6692 (9.40%)	13,574 (17.10%)	
Student or volunteer		916 (1.30%)	1474 (1.90%)	
Housekeeper or retired		20,928 (29.50%)	17,776 (22.40%)	
Unemployed due to health		35,155 (49.60%)	39,375 (49.60%)	
Unemployed not due to health		7226 (10.20%)	7266 (9.10%)	
**Severity of impairment**				<0.001
Mild		29,398 (41.00%)	34,725 (43.20%)	
Moderate		23,848 (33.20%)	25,480 (31.70%)	
Severe		9258 (12.90%)	10,543 (13.10%)	
Extreme		9232 (12.90%)	9690 (12.00%)	

Notes: The severity of impairment is determined based on the highest qualifier of body functions (b codes) and structural components (s codes) of the ICF (1 = mild: 5%–24% impairment, 2 = moderate: 25%–49% impairment, 3 = severe: 50%–95% impairment, 4 = complete: 96%–100% impairment); * Independent *t*-test.

### 3.2. WHODAS 2.0 Scores and Causes of Disability

Table 2 lists the domain and summary scores of WHODAS 2.0 according to the causes of disability. The summary scores of WHODAS 2.0 were higher in individuals with stroke, dementia, and spinal cord injury than other groups. Conversely, the scores were the lowest in the case of individuals with hearing impairment. The six domain scores of individuals with distinct types of impairment exhibited unique patterns. For example, individuals with schizophrenia received high scores in Domain 1 (understanding and communication), Domain 4 (getting along with others), Domain 5 (life activities), and Domain 6 (participation), but had low scores in Domain 2 (mobility) and Domain 3 (self-care). Individuals with stroke, dementia, and spinal cord injury received high scores across all six domains.

### 3.3. WHODAS 2.0 Scores and Severity of Impairment

**Figure 2:**
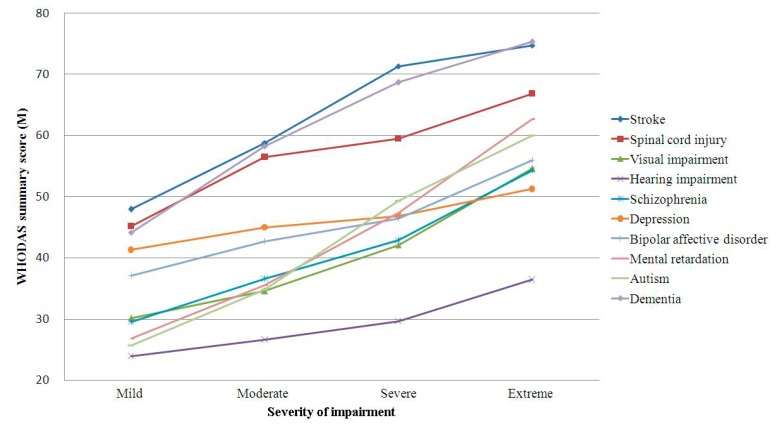
Effect of severity of impairment on WHODAS 2.0 scores: major disabling diseases.The severity of impairment is determined based on the highest qualifier of body functions (b codes) and structural components (s codes) of the International Classification of Functioning, Disability and Health (ICF) (mild: 5%–24% impairment, moderate: 25%–49% impairment, severe: 50%–95% impairment, complete: 96%–100% impairment).

Figure 2 presents the summary scores of WHODAS 2.0 according to severity of impairment. A linear relationship was observed between the summary scores of WHODAS 2.0 and the severity of impairment among these 10 diseases. However, the slopes of the lines showing the relationship between the scores and the severity of impairment in these diseases were distinct. The slope was highest in the case of individuals with schizophrenia and stroke, whereas it was lowest in the case of individuals with hearing impairment. The summary scores of WHODAS 2.0 of individuals with extreme severity in schizophrenia were similar to those with a moderate degree of stroke. Furthermore, the score was lower in individuals with extreme severity in hearing impairment than in individuals with mild stroke.

### 3.4. WHODAS 2.0 Scores and Associated Factors

Table 3 presents the relationship between WHODAS 2.0 scores and associated factors that were analyzed using a Poisson regression model. All of the associated factors were independent variables of disability; these variables were sex, age, area of residence, work status, and type and severity of impairment. Among these, work status and severity and type of impairment were major predictors of disability that had a high odds ratio.

**Table 2:** Domain and summary scores of WHODAS 2.0 according to causes of disability **(n = 101,808)**.

	Cause	Norm (n = 1507)	Schizophrenia (n = 24,602)	Hearing impairment (n = 17,361)	Stroke (n = 15,626)	Dementia (n = 13,400)	Bipolar affective disorder (n = 12,582)	Visual impairment (n = 7099)	Mental Retardation (n = 4458)	Depression (n = 4646)	Spinal cord injury (n = 1234)	Autism (n = 800)
Domain												
Domain 1		5.9 ± 11.6	35.23 ± 24.97	25.61 ± 21.66	50.10 ± 34.04	70.07 ± 27.24	37.77 ± 24.24	24.03 ± 23.83	41.93 ± 25.31	40.60 ± 23.65	29.19 ± 31.18	37.60 ± 25.61
Domain 2		3.4 ± 10.7	13.03 ± 19.58	16.37 ± 22.54	62.42 ± 31.23	53.96 ± 33.87	21.01 ± 24.15	28.60 ± 26.32	10.33 ± 18.75	23.92 ± 24.24	66.79 ± 30.53	7.24 ± 15.12
Domain 3		0.6 ± 4.7	9.99 ± 16.97	6.53 ± 15.19	44.02 ± 35.48	40.58 ± 35.19	11.21 ± 18.35	15.43 ± 22.16	11.71 ± 18.89	11.24 ± 17.91	39.53 ± 35.94	11.51 ± 19.18
Domain 4		3.5 ± 9.8	42.85 ± 28.89	35.97 ± 28.50	57.70 ± 34.55	70.84 ± 29.82	46.82 ± 28.58	30.81 ± 29.12	44.70 ± 29.68	51.43 ± 27.00	44.00 ± 34.31	51.24 ± 28.83
Domain 5-1		3.6 ± 13.4	36.47 ± 31.43	22.81 ± 31.10	75.66 ± 35.09	77.02 ± 33.64	39.46 ± 31.66	45.79 ± 36.58	40.16 ± 31.47	42.28 ± 31.00	73.53 ± 36.49	37.13 ± 32.79
Domain 5-2		1.9 ± 7.7	72.50 ± 42.18	23.76 ± 40.49	55.37 ± 49.35	35.89 ± 47.88	68.18 ± 43.42	47.24 ± 48.08	56.17 ± 44.35	70.38 ± 42.64	65.76 ± 46.54	50.97 ± 41.58
Domain 6		15.2 ± 16.0	36.11 ± 22.74	27.27 ± 21.21	56.97 ± 26.44	50.33 ± 26.11	45.59 ± 23.96	38.18 ± 24.09	31.71 ± 23.24	49.56 ± 22.40	58.22 ± 26.59	33.36 ± 23.76
Summary		6.4 ± 8.6	36.04 ± 18.90	25.36 ± 18.44	60.20 ± 23.82	61.42 ± 23.32	40.38 ± 19.87	34.73 ± 20.75	34.57 ± 20.32	43.40 ± 18.60	55.14 ± 22.61	33.10 ± 20.15

Notes: Domain 1: Understanding and communicating; Domain 2: Getting around; Domain 3: self-care; Domain 4: getting along with others; Domain 5-1: life activities, domestic; Domain 5-2: life activities, work; Domain 6: participation in society.

**Table 3:** Poisson regression of WHODAS 2.0 scores and associated variables.

Variables		B	Wald chi-square	Exp(B)
Intercept		2.268	607,427.8	9.660 *
Age		0.009	75,481.9	1.009 *
Sex				
	Male (Reference)			
	Female	0.021	431.1	1.021 *
Residence				
	Urban (Reference)			
	Suburban	0.002	3.3	1.002
	Rural	0.012	108.2	1.012 *
Work status				
	Employed (Reference)			
	Student or volunteer	0.144	708.8	1.155 *
	Housekeeper or retired	0.318	16,934.6	1.374 *
	Unemployed due to health	0.561	66,313.4	1.753 *
	Unemployed not due to health	0.280	11,156.6	1.323 *
Types of impairment				
	Sensory (Reference)			
	Mental	0.414	76,282.9	1.513 *
	Physical	0.587	128,220.5	1.798 *
Severity of impairment				
	Mild (Reference)			
	Moderate	0.127	11,252.0	1.135 *
	Severe	0.263	31,540.5	1.301 *
	Extreme	0.429	56,182.7	1.535 *

Notes: * *P* < 0.001; Types of impairment: Sensory (hearing impairment, visual impairment), Mental (schizophrenia, dementia, bipolar affective disorder, mental retardation, depression, autism), and Physical (stroke, spinal cord injury).

## 4. Discussion and Conclusions

This is one of the first studies to examine the association between a variety of impairment types and disability. Our study has shown that disability is associated with numerous variables that were identified in this study based on distinct perspectives. The data support the framework of the ICF, which holds that multiple factors interact and that all of these factors must be considered to effectively and comprehensively assess disability. Before the ICF was promulgated, the definition of disability was unclear and was debated, and thus studying the prevalence and the risk factors of disability was a major challenge. This study provides data that enhance our understanding of the relationship between various aspects of functioning by way of the ICF and health condition. Based on these findings, policy makers may need to consider the support that people with different types and levels of disabilities need to live independently, and allocate social-welfare resources to benefit people with disabilities who require the greatest assistance.

The ICF system provides a robust classification system for collecting statistics on people with disabilities [9]. WHODAS 2.0 has been used to measure the everyday functioning of individuals across six domains that correspond to the activities and participation components of the ICF. WHODAS 2.0 was designed as a generic measurement tool that is suitable for use across distinct health conditions, countries, and cultures. WHODAS 2.0 offers features that are more favorable than those offered by other validated participation measures in that no other measure presents subscale structures that directly match the components of the ICF [22]. The psychometric properties of WHODAS 2.0 have been evaluated in the case of numerous clinical conditions and found to have good validity and reliability [15,23]. Although WHODAS 2.0 has been used in several studies to assess the everyday functioning of participants, few studies have considered all of the diseases examined in this study collectively and concurrently compared with the disabilities that diseases are associated with [24].

Disability disproportionately affects vulnerable populations, particularly women, the elderly, and the poor [25]. This statement is supported by the results of this study: the odds ratios measured for females, elderly people, and individuals of a low socioeconomic status were higher than those of other individuals. Females were reported to face greater challenges than males do in social participation [26]. In Taiwan, gender differences have been decreased as a result of the efforts of the civil service and the government. Nevertheless, additional effort must be devoted toward helping the vulnerable population to reduce the challenges they face in daily lives by providing them, for example, with pensions, assistive devices, housing, and vocational rehabilitation. Further investigations may be needed to explore the relationships between these potentially influential factors and disability. For example, the interaction between work status and gender may need to be included as studying the determinants of disability. Environmental factors such as physical living environment and social stigma should also be considered in future investigations.

Our findings show that people with dementia and stroke received the highest scores in WHODAS 2.0 Domain 1 (understanding and communicating), whereas people with spinal cord injury, and hearing impairment, and visual impairment received the lowest scores. In individuals whose scores were high in the domain of understanding and communicating, social welfare such as day care center for individuals with dementia, long-term care for individuals with mental illness, care-consultation services, and support for caregivers must be provided to improve the quality of care available to the individuals [27].

Sousa *et al.* reported that the WHODAS score was the highest in dementia and cognitive impairment are the strongest predictors of disability [12]. Sousa *et al.* also stated that psychiatric disorders and stroke constitute a key independent contributor to dependency. Agreed with Sousa *et al.*’s findings [12], our results indicated that people with dementia reported the highest disability score among all impairment types.

Individuals with dementia, stroke, and SCI experienced greater challenges in activities and social participation than did other groups. While dementia and stroke have been identified as a critical determinant of dependency and hindered social participation, intervention studies are in need to improve the cognitive and mobility functions of individuals with dementia and stroke to prevent the deterioration of their daily activities and social participation.

Our results demonstrated that people with physical disabilities (stroke and spinal cord injury) received the highest scores in Domain 2 (ambulating). The requirements of these individuals are distinct from those of individuals with mental illnesses. Rehabilitation, assistive devices, wheelchairs, barrier-free environments, and public-transportation services are required to enhance the mobility function of people with physical disabilities [28]. People with stroke, dementia, and spinal-cord injury received the highest scores in Domain 3 (self-care), which indicates that rehabilitation interventions and supports such as part-time or full-time assistants, assistive devices, and barrier-free environments may be needed for these populations with their everyday activities. People with stroke, dementia, depression, and autism received the highest scores in Domain 4 (getting along with others) and Domain 5-1 (life activities). That is, social support may be needed for these groups to enhance their social participation. In this study, most of the individuals received a high score in Domain 5-2 (life activities, work). Work is a major concern in people with all types of impairment; however, most of the participants in this study did not report the challenges they faced at work. This could be explained based on the distinct cultural backgrounds of the participants and also on what must be done to advance our evaluation system. In Taiwan, people do not have to work when they are determined to have disability; thus, work is not a key concern for these individuals, and they are more concerned about their daily life and mobility than about seeking employment. In recent years, the Taiwanese government has enacted a protection law to help people with disability find jobs. The law requires private companies to hire people with disabilities to make up at least 1.5% of their work force and government agencies to hire people with disabilities to make up at least 3% of their work force. This law reflects distinct attitudes toward work exhibited in western and eastern countries. Vocational rehabilitation and job redesign should be provided by government or hospitals to help people return to work.

Korff *et al.* used WHODAS 2.0 to study the potentially modifiable factors that are associated with disability in diabetes individuals. In their study, the authors used a WHODAS 2.0 score of ≥45 as indicators of substantial disability [29]. This score is also a suitable cut-off point in all of the causes of disability examined in this study, except hearing impairment, visual impairment, mental retardation, and autism. Thus, in the future, a distinct cut-off point must be established for disabilities caused by various types of impairment as well as establishing a normative data on WHODAS 2.0.

The limitations of this study are as follows: first, this was a cross-sectional study rather than a cohort study, and thus we could not explore causal effects. Second, there were numerous evaluators conducting the evaluation of WHODAS2.0. Nonetheless, the on-job training was provided to all of the evaluators and helped them maintain a consistent evaluation procedure. Third, people with co-existing diseases were not included in this study. Therefore, we could not analyze the effect of comorbidities. Fourth, the number of participants does not represent the true prevalence of disability because the data presented here are the first-year data of a 5-year survey. We will closely monitor the prevalence of disability based on the number of all of people with disability who are included in the registry by 2019.

In public health, it is important that resources are allocated appropriately by governmental organizations to support people with different types and levels of impairment to live in society. By determining the severity of impairment and acknowledging the different contributing factors to disability, we are able to better tailor public health interventions such as community based rehabilitation and guide policy-maker to remove environmental barriers and the implementation of factors that facilitate activity and participation.

## References

[1] Officer A., Groce N.E. (2009). Key concepts in disability. Lancet.

[2] (2011). World Report on Disability.

[3] Alleyne G., Binagwaho A., Haines A., Jahan S., Nugent R., Rojhani A., Stuckler D. (2013). Embedding non-communicable diseases in the post-2015 development agenda. Lancet.

[4] Kostanjsek N., Good A., Madden R.H., Ustun T.B., Chatterji S., Mathers C.D., Officer A. (2013). Counting disability: Global and national estimation. Disabil. Rehabil..

[5] McDonald K.E., Raymaker D.M. (2013). Paradigm shifts in disability and health: Toward more ethical public health research. Amer. J. Public Health.

[6] Krahn G., Campbell V.A. (2011). Evolving views of disability and public health: The roles of advocacy and public health. Disabil. Health J..

[7] Leonardi M., Bickenbach J., Ustun T.B., Kostanjsek N., Chatterji S. (2006). The definition of disability: What is in a name?. Lancet.

[8] (2009). Disability: Beyond the medical model. Lancet.

[9] (2001). International Classification of Functioning, Disability and Health (ICF).

[10] Centers For Disease Control and Prevention CDC Grand Rounds: Public Health Practices to Include Persons with Disabilities. http://www.cdc.gov/mmwr/preview/mmwrhtml/mm6234a3.htmf.

[11] Lopez A.D., Murray C.J., Organization W.H. (1996). The Global Burden of Disease: A Comprehensive Assessment of Mortality and Disability from Diseases, Injuries, and Risk Factors in 1990 and Projected to 2020.

[12] Sousa R.M., Ferri C.P., Acosta D., Albanese E., Guerra M., Huang Y., Jacob K.S., Jotheeswaran A.T., Rodriguez J.J., Pichardo G.R. (2009). Contribution of chronic diseases to disability in elderly people in countries with low and middle incomes: A 10/66 dementia research group population-based survey. Lancet.

[13] De Pedro-Cuesta J., Alberquilla A., Virues-Ortega J., Carmona M., Alcalde-Cabero E., Bosca G., Lopez-Rodriguez F., Garcia-Sagredo P., Garcia-Olmos L., Salvador C.H. (2011). ICF disability measured by WHODAS II in three community diagnostic groups in Madrid, Spain. Gac. Sanit..

[14] Garin O., Ayuso-Mateos J.L., Almansa J., Nieto M., Chatterji S., Vilagut G., Alonso J., Cieza A., Svetskova O., Burger H. (2010). Validation of the “World Health Organization Disability Assessment Schedule, WHODAS-2” in patients with chronic diseases. Health Qual. Life Outcomes.

[15] Kucukdeveci A.A., Kutlay S., Yildizlar D., Oztuna D., Elhan A.H., Tennant A. (2013). The reliability and validity of the world health organization disability assessment schedule (WHODAS-II) in stroke. Disabil. Rehabil..

[16] Kulnik S.T., Nikoletou D. (2014). WHODAS 2.0 in community rehabilitation: A qualitative investigation into the validity of a generic patient-reported measure of disability. Disabil. Rehabil..

[17] Wolf A.C., Tate R.L., Lannin N.A., Middleton J., Lane-Brown A., Cameron I.D. (2012). The World Health Organization disability assessment scale, WHODAS II: Reliability and validity in the measurement of activity and participation in a spinal cord injury population. J. Rehabil. Med..

[18] Chiu W.T., Yen C.F., Teng S.W., Liao H.F., Chang K.H., Chi W.C., Wang Y.H., Liou T.H. (2013). Implementing disability evaluation and welfare services based on the framework of the international classification of functioning, disability and health: Experiences in Taiwan. BMC Health Serv. Res..

[19] Teng S.W., Yen C.F., Liao H.F., Chang K.H., Chi W.C., Wang Y.H., Liou T.H. (2013). Evolution of system for disability assessment based on the international classification of functioning, disability, and health: A Taiwanese study. J. Formos. Med. Assoc..

[20] Chi W.C., Liou T.H., Wennie Huang W.N., Yen C.F., Teng S.W., Chang I.C. (2013). Developing a disability determination model using a decision support system in taiwan: A pilot study. J. Formos. Med. Assoc..

[21] Yen C.F., Hwang A.W., Liou T.H., Chiu T.Y., Hsu H.Y., Chi W.C., Wu T.F., Chang B.S., Lu S.J., Liao H.F., Teng S.W. (2014). Validity and reliability of the functioning disability evaluation scale-adult version based on the WHODAS 2.0—36 items. J. Formos. Med. Assoc..

[22] Üstün T.B., Kostanjsek N., Chatterji S., Rehm J. (2010). Measuring Health and Disability: Manual for WHO Disability Assessment Schedule (WHODAS 2.0).

[23] Zhao H.P., Liu Y., Li H.L., Ma L., Zhang Y.J., Wang J. (2013). Activity limitation and participation restrictions of breast cancer patients receiving chemotherapy: Psychometric properties and validation of the chinese version of the WHODAS 2.0. 0. Qual. Life Res..

[24] Almazan-Isla J., Comin-Comin M., Damian J., Alcalde-Cabero E., Ruiz C., Franco E., Martin G., Larrosa-Montanes L.A., de Pedro-Cuesta J. (2014). Analysis of disability using WHODAS 2.0 among the middle-aged and elderly in Cinco Villas, Spain. Disabil. Health J..

[25] Hosseinpoor A.R., Stewart Williams J.A., Gautam J., Posarac A., Officer A., Verdes E., Kostanjsek N., Chatterji S. (2013). Socioeconomic inequality in disability among adults: A multicountry study using the world health survey. Amer. J. Public Health.

[26] Subramaniam M., Abdin E., Vaingankar J.A., Chong S.A. (2013). Gender differences in disability in a multiethnic Asian population: The Singapore mental health study. Compr. Psychiat..

[27] Yen C.F., Chiu T.Y., Liou T.H., Liao H.F., Li Y.S., Liang C.C., Teng S.W. (2014). Does the planned long-term care policy in Taiwan meet the needs of people with disabilities?. Health Policy.

[28] Borg J., Lindstrom A., Larsson S. (2009). Assistive technology in developing countries: National and international responsibilities to implement the convention on the rights of persons with disabilities. Lancet.

[29] Von Korff M., Katon W., Lin E.H., Simon G., Ludman E., Oliver M., Ciechanowski P., Rutter C., Bush T. (2005). Potentially modifiable factors associated with disability among people with diabetes. Psychosom. Med..

